# Editorial: Ethnopharmacology of the lamiaceae: Opportunities and challenges for developing new medicines

**DOI:** 10.3389/fphar.2022.961486

**Published:** 2022-08-25

**Authors:** Sujogya Kumar Panda, Luc Van Puyvelde, Marie Jeanne Mukazayire, Zilda Cristiani Gazim

**Affiliations:** ^1^ Centre of Environment Climate Change and Public Health, RUSA, Utkal University, Bhubaneswar, India; ^2^ Department of Biology, Animal Physiology and Neurobiology Section, KU Leuven, Leuven, Belgium; ^3^ Department of Pharmacy, School of Pharmacy and Medicine, College of Medicine and Health Sciences, University of Rwanda, Huye, Rwanda; ^4^ Graduate Program in Animal Science and Biotechnology Applied to Agriculture, Chemistry Laboratory of Natural Products, Paranaense University, Umuarama, Brazil

**Keywords:** lamiaceae, drug discovery, phytochemistry, cardio vascular diseases, antiapoptosis, anti-hyperalgesic, antioxidant

The Lamiaceae (also known as the mint family) is one of the largest botanical families, with 6,900–7,200 accepted species names, under 236 genera. The importance of the family for medicinal, aromatic, environmental, and food purposes has been known for centuries. Species in the mint family are recognized in traditional medicine throughout the world. Some particularly prominent genera include *Salvia* (900 spp.), *Scutellaria* (360 spp.), *Stachys* (360 spp.), *Hyptis* (280 spp.), and *Thymus* (360 spp.). Many of the species are cultivated including several widely used culinary botanical drugs viz. *Ocimum basilicum* L.; *Lavandula angustifolia* Mill.; *Origanum majorana* L.; *Mentha* L.; *Origanum vulgare* L.; *Rosmarinus officinalis* L.; *Salvia officinalis* L.; *Satureja hortensis* L. and *Thymus vulgaris* L.

The focus of this research topic is to make a special contribution to the ethnopharmacology of the species that make up the Lamiaceae family; considering the phytochemistry and molecular pharmacology of its secondary metabolites. All the manuscripts that make up this edition show the importance of Lamiaceae and their secondary metabolites in the prevention and treatment of various diseases; evidencing that many drugs available today have their origin in plant molecules.

A very important topic in this issue assesses the influence of seasonality on the chemical composition and biological activities of species that make up the Mentha genus, common to the Mediterranean and subtropical regions, and has received considerable attention for being a rich source of a variety of phenolic compounds and also for its numerous therapeutic properties (Abbou et al., 2022). However, the variability and alteration of phytochemical composition due to different ecological and geographical factors can lead to differences in biological activities and pharmaceutical properties of the plant (Mollaei et al., 2020). Brahmi et al. studied the influence of environmental factors on the levels of phenolic compounds of three species of the genus Mentha (*M. pulegium, M. rotundifolia,* and *M. spicata*) from different regions of Algeria. The authors, based on phytochemical analyses, conclude that the phenolic content observed highest in distinct regions, probably due to environmental factors such as soil nature, altitude, temperature, and precipitations, conjugated with eventual region-dependent biotic factors. Also, variation in antioxidant capacity was observed according to geographical and ecological factors. The results obtained in this study allowed determining the best local conditions for the growth of the studied species for a higher concentration of phenolic compounds and their possible use in different fields such as health care and food. Nevertheless, more research is needed to precise the causes of location effects, extend the study over several years, determine the heritability of phenolic compounds and antioxidant properties, and develop a production system that ensures exploitable biomasses.

Menthol (the key monoterpene found in *Mentha × piperita* L.) is known to modulate the nociceptive threshold and is present in different curative preparations that reduce sensory hypersensitivities in pain conditions. Pulegone is another similar type of compound present in *Calamintha nepeta* used in traditional medicine to alleviate rheumatic disorders, a chronic inflammatory disease. With advancements in gas chromatography-mass spectrometry, Hilfiger et al. studied *in vitro* anti-inflammatory activities of menthol and pulegone by measuring the secretion of the tumor necrosis factor-alpha (TNF α) from LPS-stimulated THP-1 cells. Furthermore, both menthol and pulegone have been tested for *in vivo* anti-hyperalgesic effects with a rat inflammatory pain model. *In vivo* rat model study showed pulegone to exert a higher transient anti-hyperalgesic effect on the nociceptive sensory thresholds than menthol; the authors compared the impacts of menthol and pulegone on inflammation and pain. Interestingly, both compounds act as strong anti-inflammatory and anti-hyperalgesic monoterpenes. Authors claimed that pulegone and menthol are the most abundant monoterpenes found in *C. nepeta* (49.41%) and *M. piperita* (42.85%) extracts, respectively. This is the first report of pulegone as anti-hyperalgesic suggesting that traditional treatments based on *C. nepeta* and *M. piperita* preparations have indeed the potential to reduce pain and inflammation. Further studies comparing the toxicity of pulegone and menthol at the therapeutic dose and their in-depth mechanisms of action will be required to assess their potential as drug candidates.

In a very elegant study, Berdowska et al. assessed the modulatory impact of Lamiaceae phytoconstituents on the apoptosis of human leukemia cells. The authors evaluated the impact of some dried aqueous extracts (*Thymus vulgaris* L., *Thymus serpyllum* L., *Origanum majorana* L., and *Mentha × piperita* L.) along with known phenolic compounds (caffeic acid, rosmarinic acid, lithospermic acid, luteolin-7-O-β-glucuronide, luteolin-7-O-rutinoside, eriodictyol-7-O-rutinoside, and arbutin) on both unstimulated and staurosporine-stimulated Jurkat cells. The results suggested the pro-apoptotic actions against human leukemia Jurkat cells of polyphenolic mixtures derived from *T. serpyllum* and *O. majorana*, enhancement of staurosporine-induced apoptosis by both caffeic and rosmarinic acids, and less evident pro-apoptotic effects of purified (poly)phenolic compounds. Nevertheless, due to the limitation of the present study, if longer incubation time and greater concentrations of the tested compounds had been applied, stronger effects might have been observed. Therefore, the present experiments may be considered rather as a pilot study to encourage their continuation with the aim to gain more definite conclusions.

*Salvia* is another significant genus of the Lamiaceae that is used worldwide for its medicinal and culinary uses. Most of the ethnopharmacological and phytochemical studies on Salvia are focused on species from the Asian and European clades. Hence, studies on the Neotropical sages (*Salvia* subgenus Calosphace; 587 spp.), are comparatively sporadic. Ortiz-Mendoza et al. compiled on the traditional uses, pharmacological and phytochemistry properties of the Mexican Neotropical Sages. The most common occurrences of active compounds are composed of the diterpenes, particularly clerodanes (e.g., Amarisolide A, Tilifodiolide). Research on these metabolites guided by the phylogenies is recommended since closely related species tend to share the presence of similar compounds and thus similar medicinal properties.

Selected members of the Lamiaceae family are widely used in traditional medicine to cure various disorders with some as potent cardioprotective plants (Patrignani et al., 2021). One of the best examples of a plant currently being used in developing a new hypertension drug is *Marrubium vulgare* L. The crude oil of *M. vulgare* was examined for hypotensive effect, which was scientifically proven due to the presence of diterpenoids (Michel et al., 2020).

And in this sense, Chakrabartty et al. performed an extensive literature review of members of Lamiaceae with antioxidant, anti-hyperlipidemic, vasorelaxant, and thrombolytic effects. The authors described different contributions of plants to different aspects of cardiovascular diseases like stroke, heart attack, etc. evidencing those phenolic acids, flavonoids, alkaloids, and other phytochemicals are responsible for these actions. Another point addressed in this research is functional foods, revealing that these plants are a rich source of nutrients and minerals such as omega-3, which may have an additional role in the prevention of cardiovascular diseases. Furthermore, the authors conclude that limitations still exist and that extensive research needs to be carried out on the Lamiaceae family in the quest to develop new and effective botanical drugs as well as functional foods that can be used in the treatment and prevention of diseases. cardiovascular diseases worldwide.

The rapid rising of antimicrobial resistance and the need for new approaches to fill the gap in antimicrobial drug discovery are alarming (Jouneghani et al., 2020). *Tetradenia riparia* Hochsteter codd. A plant belonging to Lamiaceae is being used to treat several bacterial and fungal infections. This species has been researched for decades to isolate and identify several chemical constituents such as ibozol, 7α-hydroxyroileanone, 1′,2′-dideacetylboronolide, 8 (14),15-sandaracopimaradiene-7α,18-diol; 5,6-dihydro-α-pyrone and α-pyrone. The compound 8 (14),15- sandaracopimaradiene-7α, 18-diol has demonstrated multiple bioactivities, such as antispasmodic, anthelmintic, and antimicrobial (foodborne pathogens, MDR *Staphylococcus* and *Mycobacterium*). Biofilm-producing bacteria are typically much less sensitive to antimicrobials than planktonic cells (Sahoo et al., 2021). Interestingly, the extract of *T. riparia* had strong effects against pre-formed *S. aureus* biofilm with clear disruption of organized structure of *S. aureus* ATCC 29213 biofilms under SEM (Endo et al., 2018). Moreover, Costa et al. (2015) found that a hydroalcoholic extract of *T. riparia* inhibited *C. albicans* biofilm which was more effective compared with standard fluconazole. Extract as well as the compound 8 (14),15-sandaracopimaradiene-7α,18-diol were found to be bactericidal against *S. aureus*, with antibiofilm activity (BIC_50_, 8.8 ± 1.5 μg/ml) (Van Puyvelde et al., 2021). Moreover, Endo et al. (2018) combined *the* hydroalcoholic extract along with penicillin against a panel of MDR *S. aureus* strains and found synergistic effects. A similar observation was also observed by Costa et al. (2015) when combined with nystatin against *C. albicans.* Other major bioactive compounds are 6,7-dehydroroyleanone (antimicrobial, antiparasitic), ibozol (antimicrobial, antitumor), and abieta-7,9 (11)-dien-13-β-ol (antimicrobial) was also testified from several experimental investigations.

The present research topic thus includes interdisciplinary research work expanding the knowledge about traditional uses of Lamiaceae fostering the scientific field of ethnopharmacology globally. This research topic successfully gathered comprehensive information in the field of drug discovery related to *hyperalgesia,* cardiovascular diseases, and cancer. Additional work is needed to isolate and characterize the active compounds and further follow–up studies are also necessary to elucidate the mechanism of action and SAR. Moreover, pharmacokinetic and toxicity studies will be required to assess their potential as drug candidates.

In view of what has been exposed in this special issue, we understand that despite the discovery of many compounds with high biological potential, further studies are needed to assess their toxicity as well as clinical studies. The limitation of this special issue is that the published papers are mostly focused on ethnopharmacology (regional, traditional, or cross-cultural practices), qualitative and quantitative composition of secondary metabolites, and identification of bioactive compounds (GC-MS, LC-MS, NMR) but lack studies on the development of molecular targets for drug discovery, randomized controlled clinical trials on the efficacy and safety of any Lamiaceae plants, either alone or in combination with western medicine. Moreover, most of the original research focuses on essential oil while follow-up with bioassay-guided purification is lacking, which needs to be followed up specially from the genera that are insufficiently explored. Many of the members of the Lamiaceae family often become matters for scientific validation but are not yet much explored, and scientists must give attention to their study in the near future.

We would like to thank all the reviewers for their comments that improved our manuscripts, and the authors for their excellent contributions.

We hope that this article collection will inspire scientists from different fields of research focusing on the assessment of traditional medicine, derived from Lamiaceae or other plant families, in the search for new pharmacological strategies.
